# The Molecular Effects of a Polymorphism in the 5′UTR of Solute Carrier Family 44, Member 5 that Is Associated with Birth Weight in Holsteins

**DOI:** 10.1371/journal.pone.0041267

**Published:** 2012-07-18

**Authors:** Mayumi Sugimoto, Toshio Watanabe, Yoshikazu Sugimoto

**Affiliations:** 1 National Livestock Breeding Center, Nishigo, Japan; 2 Shirakawa Institute of Animal Genetics, Nishigo, Japan; Goethe University, Germany

## Abstract

Dystocia is a major problem for the dairy cattle industry, and the observed high rates of this condition stem from genetic selection to increase subsequent milk production of the calving female. Because smaller birth size does not adversely affect subsequent milk production, selecting for cows with a smaller birth size would reduce dystocia rates and be beneficial for both the cattle and the farmers. To identify genes that regulate birth weight, we conducted a genome-wide association study using 1151 microsatellite markers and identified a single nucleotide polymorphism (SNP) associated with birth weight: A-326G in the 5′ untranslated region (UTR) of *solute carrier family 44, member 5* (*SLC44A5*). Cows with higher birth weights carried the A polymorphism in the *SLC44A5* 5′ UTR, and the presence of the A polymorphism correlated with a high rate of dystocia. Luciferase assays and quantitative polymerase chain reaction (QPCR) assays revealed that *SLC44A5* transcripts with the A polymorphism are expressed at lower levels than those carrying the G polymorphism. *SLC44A5* encodes a choline transporter-like protein, and choline is a component of the major phospholipids of cell membranes. Uptake studies in HeLa cells demonstrated that *SLC44A5* knockdown reduces choline efflux, whereas *SLC44A5* overexpression resulted in the opposite effect. Furthermore, cell viability assays indicated that *SLC44A5* knockdown increased cell proliferation, whereas *SLC44A5* overexpression repressed proliferation. Taken together, our results suggest that calves with reduced *SLC44A5* expression are larger due to enhanced cell proliferation. This study provides novel insights into the molecular mechanisms that control birth weight in Holsteins and suggests that SLC44A5 may serve as a potential target for preventing dystocia.

## Introduction

Dystocia has a major economic impact on the dairy cattle industry. One study estimated that the cost of dystocia with extremely difficult labor was nearly $400 per incident [1]. Selective breeding has resulted in larger cows that have a higher milk production potential, but these larger cows also tend to induce dystocia in the calving female [2]. The probability of dystocia increases by 13% for each kg increase in birth weight [3]. Moreover, high milk production in the dam predisposes it to give birth to a smaller calf, and a lower birth size does not have any subsequent adverse effects on milk productivity [4]. Therefore, selecting for cows with a smaller birth size would prevent dystocia and be beneficial for farmers.

Whole-genome scans for quantitative trait loci (QTL) associated with birth weight or dystocia have been previously conducted [5], [6]. However, this method has identified only one gene, which encodes for non-SMC condensin I complex, subunit G, as a genetic factor that modulates fetal growth in cattle [7]. Birth weight is a quantitative trait that is controlled by many genes, and an additional whole-genome scan is warranted.

Choline is a component of the major phospholipids of cell membranes [8]. Choline deficiency reduces cell proliferation and increases apoptosis [9], suggesting that choline transporters are important for regulating cell proliferation. There are three systems for choline transport: (i) polyspecific organic cation transporters (OCTs) with low affinities for choline; (ii) high-affinity choline transporters (CHTs), and (iii) intermediate-affinity choline transporter-like (CTL) proteins [10]. Hemicholinium-3 (HC-3) is one of the strongest CHT inhibitors and has been shown to inhibit cell proliferation in human colon cancer [11], [12] and lung cancer cells [13]. It remains unclear, however, how each choline transporter is involved in proliferation.

Here, we demonstrate that cows with high birth weights carry an A polymorphism in the 5′ untranslated region (UTR) of *solute carrier family 44, member 5* (*SLC44A5*). This gene encodes a CTL protein, and the A polymorphism is correlated with an increased dystocia rate in the calving female. Luciferase assays and quantitative polymerase chain reaction (QPCR) assays reveal that the number of *SLC44A5* transcripts with the A polymorphism is reduced compared to the number of transcripts with the G polymorphism. Choline uptake studies and cell viability assays in HeLa cells further indicate that *SLC44A5* knockdown reduces choline efflux and increases cell proliferation. Our results therefore demonstrate an unexpected role for SLC44A5 in regulating birth weight.

## Results

**Figure 1:**
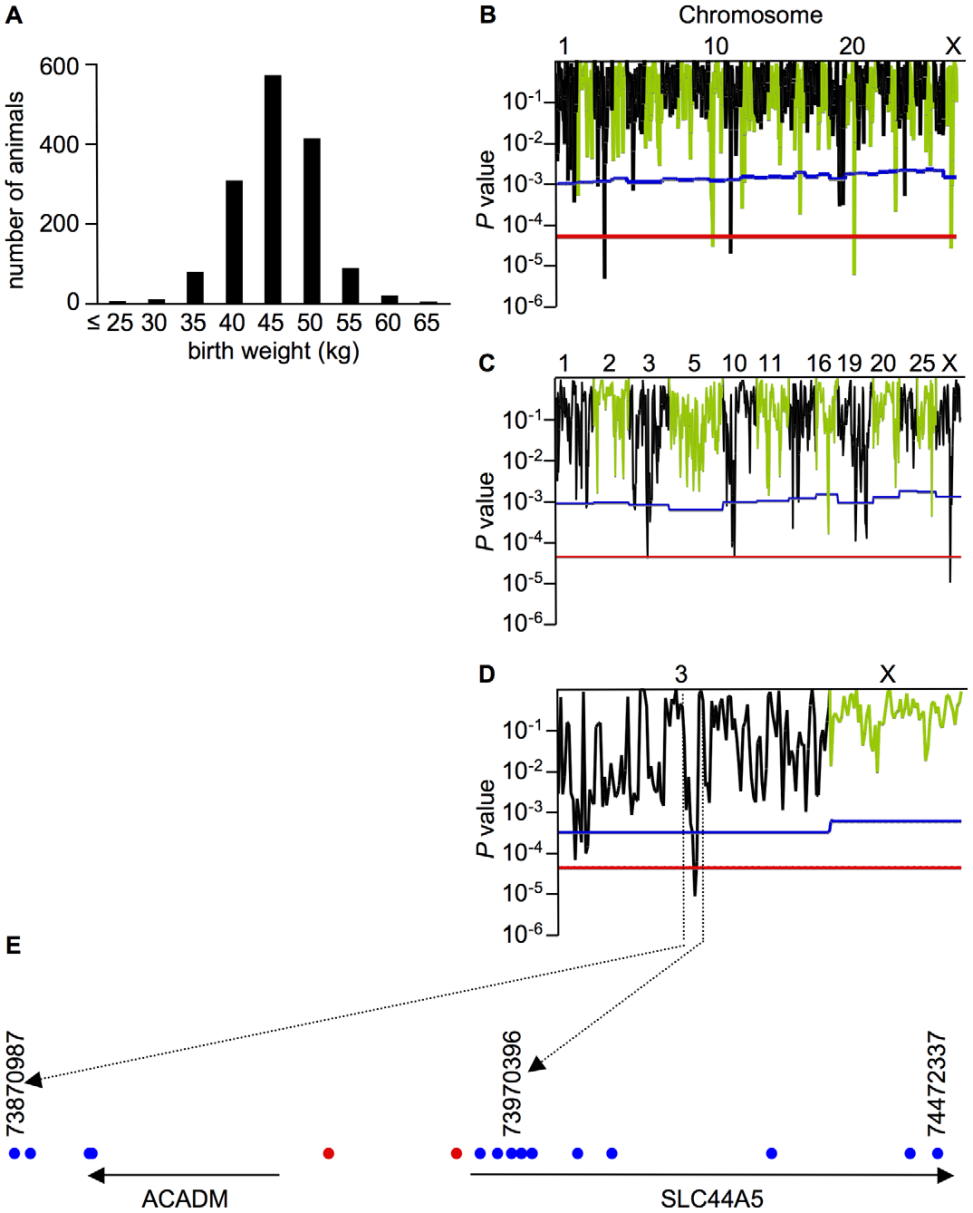
Birth weight is associated with a locus on chromosome 3. A. The distribution of birth weights among the samples. B, C, D. The association signals with birth weight for the 1^st^ (B), 2^nd^ (C), and 3^rd^ (D) screenings. The blue and red lines represent the threshold for chromosome-wide and genome-wide significance following the Bonferroni correction for multiple comparisons, respectively. E. A schematic representation of the genes (black arrow) and the microsatellite markers (blue dot) located in the critical region. The red dots represent the most significantly associated microsatellite markers. The numbers represent the positions of microsatellite markers in base pairs (bp).

To identify genes that regulate birth weight, we collected DNA from 1483 female Holstein calves and recorded their birth weight in the National Livestock Breeding Center. The birth weight of these calves ranged from 22 to 65 kg, with a median weight of 43.5 kg (Figure 1A). Of the collected samples, we selected 86 cows whose birth weight was greater than 51 kg. An equal number were selected with a birth weight of less than 35 kg. To reduce the effects of specific sires, fewer than five cows derived from the same father were included. Based on typing 1151 microsatellite markers covering from chromosomes 1 to 29 and X, the population structure of the selected samples was evaluated with STRUCTURE [14] and we found no evidence of a systematic bias (Figure S1). The stratification [15] of our samples was also low (λ = 1.0997), which suggests that there is no population structure.

We scanned a total of 172 bovine genomes and revealed a significant association at the chromosome- or genome-wide level between birth weight and markers associated with chromosomes 1, 2, 3, 5, 10, 11, 16, 19, 20, 25, and X (Figure 1B). Further analysis with an additional 111 markers showed a significant association at the genome-wide level on chromosomes 3 and X (Figure 1C). We scanned these chromosomes with an additional 179 markers and determined that the candidate genes were located in the region between 73.87 and 73.97 Mb on chromosome 3 (Figure 1D). The genes in this region included *acyl-CoA dehydrogenase, C-4 to C-12 straight chain* (*ACADM*) and *SLC44A5* (Figure 1E).

**Figure 2:**
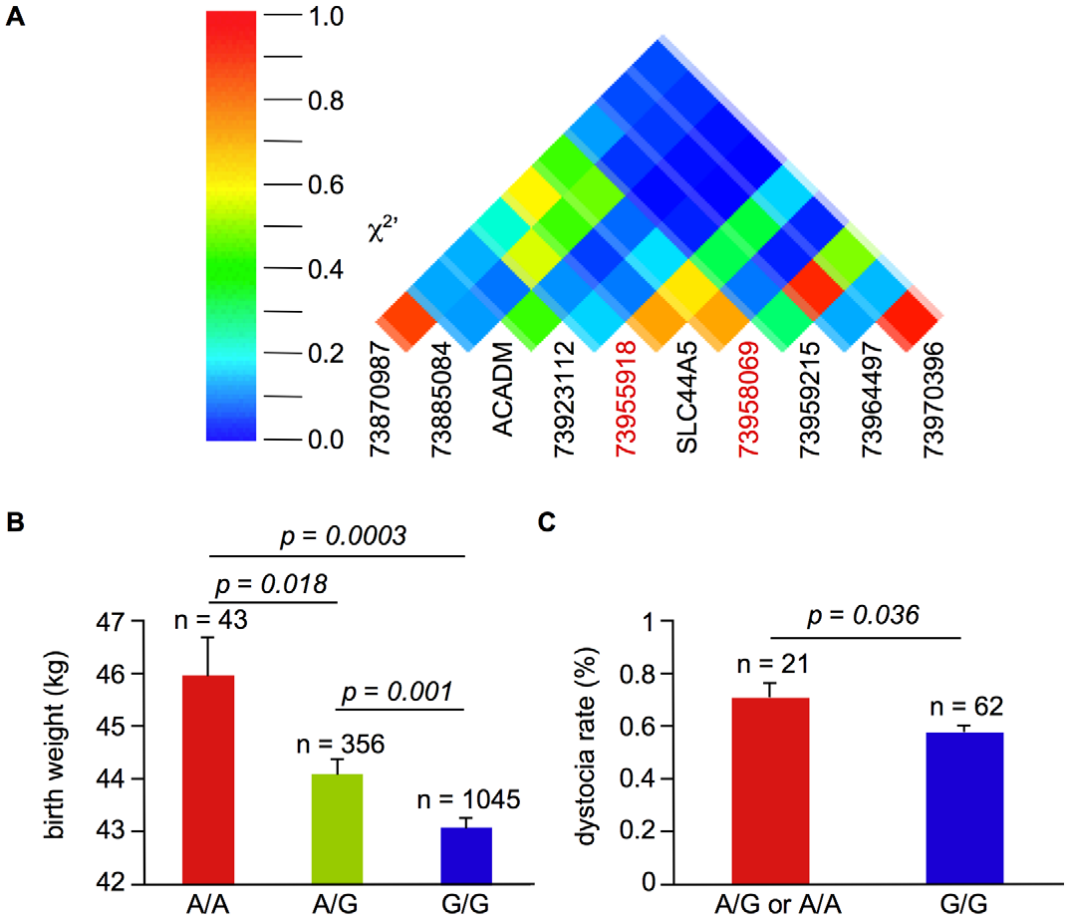
The *SLC44A5* 5′ UTR SNP is associated with birth weight. A. The LD block for the critical region in chromosome 3. The numbers represent the positions of the microsatellite markers in bp. ACADM and SLC44A5 indicate the SNPs identified in each gene. B. The average birth weight ± SE values for the calves. The *p*-value was calculated using the Student's t-test. C. The average dystocia rate ± SE values for sires. The *p*-value was calculated by the Student's t-test.

To detect potential causative polymorphisms in *ACADM* and *SLC44A5*, we sequenced each exon and the 5′ UTRs of these genes. We identified the single nucleotide polymorphisms (SNPs) T-48A and A-326G in the 5′ UTR of *ACADM* and *SLC44A5*, respectively. The SNP identified in *SLC44A5* and its neighboring microsatellite markers were in strong linkage disequilibrium (LD) with each other; the pairwise χ^2^′ measures were all greater than 0.6 (Figure 2A). The region between the neighboring microsatellite markers located at 73.956 and 73.958 Mb showed the most significant association (Figure 1D and E), and the identified SNP in *ACADM* was not included in the LD block (Figure 2A). *SLC44A5* was therefore the more promising of the two candidate genes.

To examine the effect of the *SLC44A5* SNP on the birth weight of cattle, we sequenced *SLC44A5* in the original 1483 samples. The average birth weight of 43 cows that were homozygous for the A allele was 46.0±0.7 kg, whereas the average birth weight of 1045 cows that were homozygous for the G polymorphism was 43.1±0.2 kg (Figure 2B). The difference in weight was 2.9 kg and accounted for 11% of the birth weight variation in all samples. We confirmed the same effect of the *SLC44A5* SNP on the birth weight of 1014 female Holstein calves newly collected (Figure S2A) and 389 male Holstein calves (Figure S2B). We also genotyped this polymorphism in the commercially available Holstein sires in Japan and found that the average dystocia rate of female calves derived from the 21 sires carrying the A polymorphism was 0.72±0.06%, while the average dystocia rate for daughters derived from the 62 sires that were homozygous for the G polymorphism was 0.58±0.02% (Figure 2C). These results suggested that the SNP we identified in *SLC44A5* is associated with birth weight in cattle and influences the rate of dystocia.

**Figure 3:**
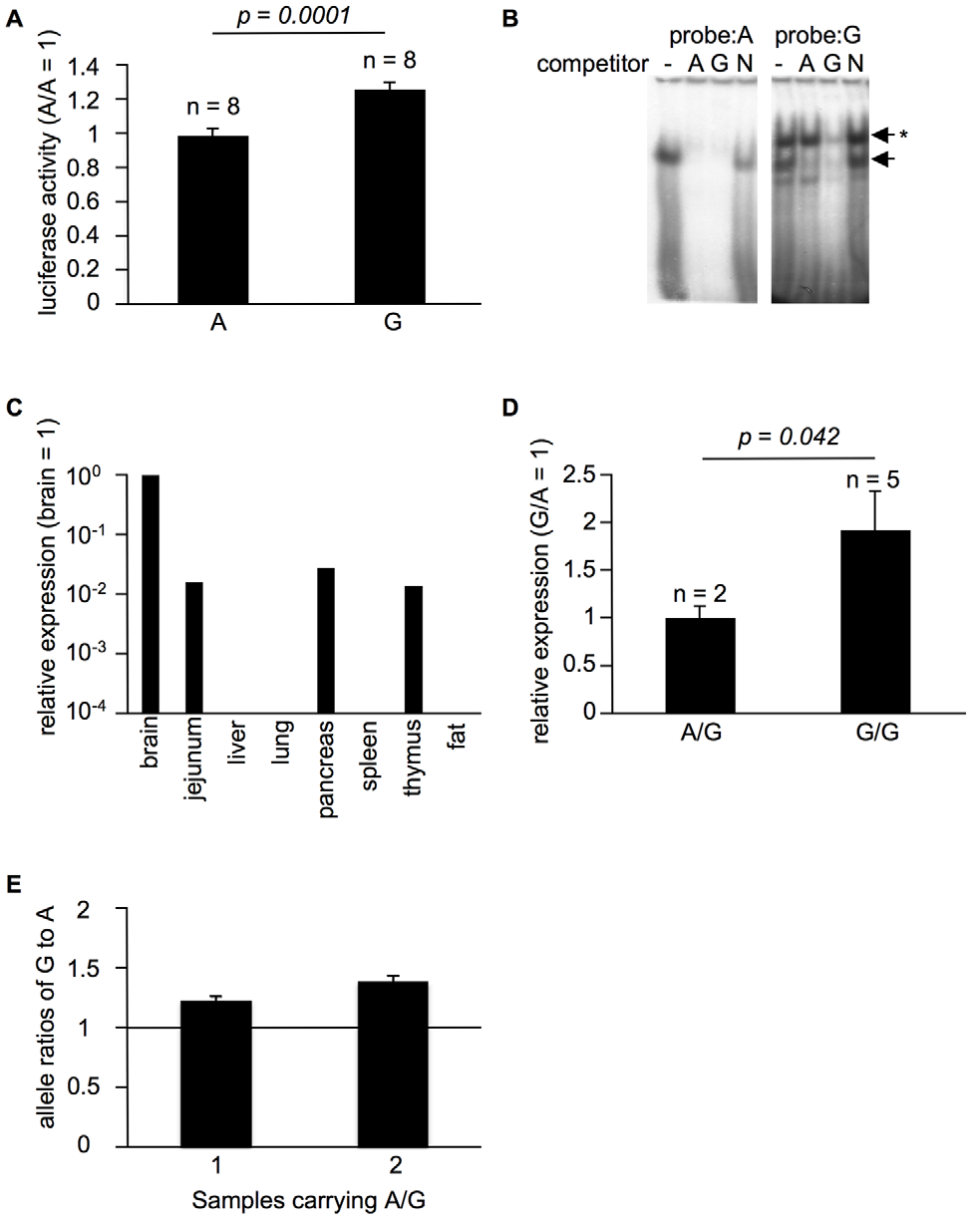
The *SLC44A5* 5′ UTR SNP controls its expression level. A. The relative luciferase activity of the 5′ UTR region of *SLC44A5*. The data are presented as the mean ± SEM. The *p*-value was calculated using the Student's t-test. B. A gel mobility shift assay of HeLa nuclear protein using the 5′ UTR region of *SLC44A5* as the probe. The binding indicated by the arrow was abolished by co-incubation with an unlabeled competitor with the A or G polymorphisms but not by a competitor containing the AP1 sequence (N). The G polymorphism-specific binding is indicated by the arrow with a star and was abolished only by co-incubation with an unlabeled competitor with the G polymorphism. C. The relative expression levels of *SLC44A5* in bovine tissues. D. The relative expression level of *SLC44A5* in the bovine brain. The data are presented as the mean ± SEM. The *p*-value was calculated using the Student's t-test. E. Average allele-specific expression level ± SE in the heterozygous bovine brain. The ratios of G to A relative to genomic DNA were shown.

The SNP in the 5′ UTR of *SLC44A5* may have an effect on the expression level of this gene. To examine whether the identified SNP affects transcriptional levels, we transfected HeLa cells with luciferase reporters carrying either of the *SLC44A5* 5′ UTR SNPs. As expected, the transfected constructs differentially affected the luciferase activity (Figure 3A); transfection with the *SLC44A5* construct carrying the A polymorphism resulted in lower luciferase activity than the construct carrying the G polymorphism, suggesting that this SNP has a biological function.

Encouraged by this finding, we performed a gel mobility-shift assay using HeLa cells. As shown in Figure 3B, a specific complex was obtained using the probe carrying the G polymorphism but not with a probe carrying the A polymorphism. Competition assays confirmed the specificity of this complex (Figure 3B). Therefore, the sequence with the G polymorphism is bound by a nuclear factor, and the presence of the probe carrying the A polymorphism abrogates this interaction.

To examine the expression levels of *SLC44A5 in vivo*, we performed QPCR and found that the bovine brain exhibits the highest expression of this gene (Figure 3C). Consistent with the results of the luciferase assay (Figure 3A), the levels of *SLC44A5* messenger RNA (mRNA) in the brains of animals carrying the A polymorphism were lower than those in brains from animals that were homozygous for the G polymorphism (Figure 3D). Even though there were only two heterozygous samples which were collected randomly at a slaughter house, we also confirmed that the level of mRNA from the G allele yielded higher than the A allele by determining the allelic mRNA ratio based on SNaPshot (Figure 3E). Taken together, these findings indicate that *SLC44A5* transcripts with the A SNP are expressed at lower levels than transcripts with the G SNP in cattle, and that lower expression levels of *SLC44A5* are associated with higher birth weights.

**Figure 4:**
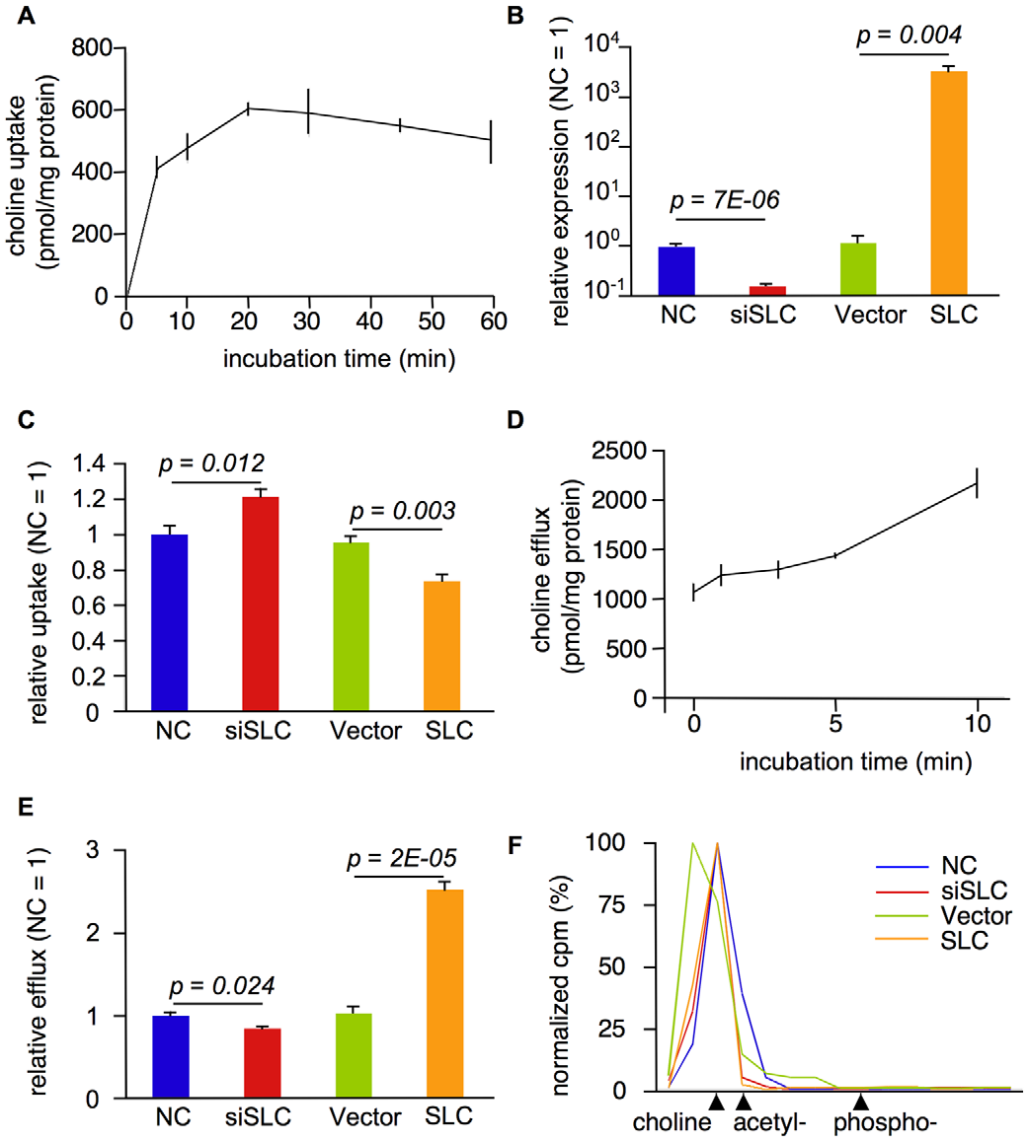
SLC44A5 increases choline efflux. A. A 60-min time course to determine the uptake of 10 nM [^3^H]choline in HeLa cells. Each point represents the uptake mean ± SEM (n = 4). B. The relative expression levels of *SLC44A5* in transfected HeLa cells. Each QPCR experiment was performed with nine replicates with samples from three transfection experiments. The data are presented as the mean ± SEM. The *p*-values were calculated using the Student's t-test. C. The relative choline uptake in transfected HeLa cells that were incubated for 20 min with 10 nM [^3^H]choline. The data are presented as the mean ± SEM (n = 4). The *p*-values were calculated using the Student's t-test. D. A 10 min time-course of choline efflux in HeLa cells following incubation for 20 min with 50 nM [^3^H]choline. Each point represents the mean ± SEM (n = 4). E. The relative choline efflux in the transfected HeLa cells following incubation for 20 min with 50 nM [^3^H]choline. The data are presented as the mean ± SEM (n = 4). The *p*-values were calculated using the Student's t-test. F. The normalized cpm of the released compounds from transfected HeLa cells following incubation for 20 min with 250 nM [^3^H]choline.

*SLC44A5* encodes a CTL protein, and the expression level of *SLC44A5* may affect the level of cellular choline uptake. To test this hypothesis, we investigated whether choline uptake occurs in normal, untransfected HeLa cells. As shown in Figure 4A, choline uptake in HeLa cells increased in a time-dependent manner and was linear up to the 20 min time-point. We then treated HeLa cells with siRNA against *SLC44A5* (siSLC) or with negative control (NC) siRNA and measured choline uptake in HeLa cells for 20 min. Compared to transfection with the NC, *SLC44A5* mRNA levels were reduced by more than 80% in HeLa cells treated with siSLC (Figure 4B). siRNA-mediated knockdown of *SLC44A5* surprisingly increased choline uptake compared to control cells (Figure 4C). Alternatively, following the transfection of HeLa cells with a bovine *SLC44A5* expression plasmid (SLC), we observed a significant reduction in choline uptake compared to HeLa cells that were transfected with an empty vector control (Vector, Figure 4B and C).

OCT2 is a low-affinity OCT and has been known to release choline from lung epithelium cells [16]. We therefore investigated whether choline release was observed in normal, untransfected HeLa cells. As shown in Figure 4D, the rate of choline release was constant in a time-dependent manner up to the 10 min time-point. We next treated HeLa cells with either *SLC44A5* siRNA or an expression plasmid and measured choline efflux for 10 min. As expected, choline efflux was decreased in *SLC44A5* siRNA-treated cells, whereas *SLC44A5* overexpression had the opposite effect (Figure 4E), suggesting that SLC44A5 may be involved in choline efflux.

Choline is a precursor of acetylcholine and phosphocholine [10]. To specifically examine what SLC44A5 is responsible for transporting, we performed chromatography on the compounds released from HeLa cells transfected with various experimental and control constructs. Metabolic studies revealed that the most CPMs were observed in the choline fractions obtained from the chromatographic separation of NC, siSLC, and SLC-treated HeLa cells (Figure 4F). The ratios of acetylcholine to choline ranged from 2–40% in the NC-, siSLC-, Vector-, and SLC-treated cells (Figure 4F). These results suggest that SLC44A5 may act as a transporter of excess choline.

**Figure 5:**
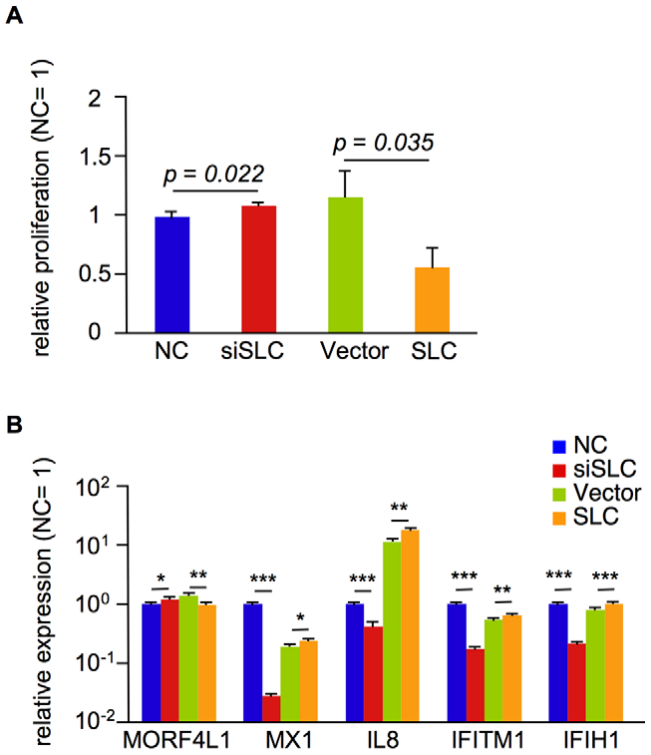
SLC44A5 suppresses proliferation. A. The relative proliferation of transfected HeLa cells. The data are presented as the mean ± SEM (n = 8). The *p*-values were calculated using the Student's t-test. B. The relative expression of genes related to proliferation and apoptosis in transfected HeLa cells. Each QPCR was performed with nine replicate with samples from three transfection experiments. The data are presented as the mean ± SEM. The *p*-values were calculated using the Student's t-test. *, **, and *** indicate *p*<0.05, *p*<0.005, and *p*<0.0005, respectively.

Choline deficiency reduces cell proliferation and increases apoptosis [9]. We investigated whether SLC44A5 suppresses cell proliferation using cell viability assays. As shown in Figure 5A, *SLC44A5* knockdown increased cell proliferation, whereas *SLC44A5* overexpression decreased proliferation. These observations suggest that *SLC44A5* expression levels influence cell proliferation and may also modulate fetal growth in cattle.

To identify the possible mechanisms by which SLC44A5 affects growth, we performed a genome-wide mRNA expression analysis of HeLa cells that were transfected with NC, siSLC, Vector, and SLC expression constructs. Using the Mann–Whitney U-test, we identified 389 probe sets were downregulated by SLC44A5 (NC < siSLC and vector > SLC; *p* = 1.3E-24), whereas 604 probe sets were upregulated by SLC44A5 (NC > siSLC and Vector < SLC; *p* = 1.3E-24; Table S1). From these probe sets, we selected several genes that are related to proliferation or apoptosis and confirmed the effects of SLC44A5 on their expression using QPCR (Figure 5B). Among these genes was *Mortality Factor 4 like 1* (*MORF4L1*), which was downregulated by SLC44A5 and is involved in both chromatin remodeling the regulation of cell proliferation [17]. *Myxovirus resistance 1, interferon-inducible protein p78* (*MX1*) is known to induce apoptosis and was upregulated by SLC44A5 [18]. Also upregulated was *Interleukin 8* (*IL8*), a suppressor of cell proliferation [19]. Moreover, *Interferon-induced transmembrane protein 1* (*IFITM1*) was upregulated by SLC44A5 and is involved in the transduction of antiproliferative and homotypic adhesion signals [20]. Lastly, *Interferon-induced with helicase C domain 1* (*IFIH1*) was also upregulated by SLC44A5 and has been shown to lead to the efficient activation of mitochondrial apoptosis [21]. Taken together, these results suggest that SLC44A5 may suppress cell growth by inducing apoptosis via the reduction of the intracellular choline level.

## Discussion

In this study, we identified a SNP in the 5′ UTR of *SLC44A5* that is correlated with birth weight in cattle and with the rate of dystocia; cows carrying the G polymorphism express this gene at higher levels. *SLC44A5* encodes a choline transporter-like protein, and our results demonstrate that *SLC44A5* overexpression suppresses cell proliferation. If farmers select for cows that carry the G polymorphism in the *SLC44A5* 5′ UTR, this would results in calves with smaller birth weights, preventing difficult labors.

Using 1151 microsatellite markers, we successfully identified the region associated with birth weight in cattle. We also narrowed the region of 0.1 Mb with additional 290 microsatellite markers (Figure 1D). Now association studies using SNPs with high density are more popular than using microsatellite markers. However, typing more than one thousand microsatellite markers could still be a useful method for association studies at least in cattle. One reason is that microsatellite markers are more polymorphic than SNPs and give more information of recombination. The other reason is that the extent of LD on cattle is greater than human [22] and less markers could be enough to identify the region in association studies for cattle. Thus it is worth typing of microsatellite markers for association studies although it is laboriousness. Recently we also identified the region associated with ovulation rate in cattle using 1154 microsatellite markers [23].

Although *SLC44A5* seems to have a major QTL effect on bovine birth weight, there are additional QTL other than this gene on chromosome 3. Heritability estimates for birth weight are 53% in a Holstein-Friesian population [24], whereas the *SLC44A5* SNP we identified accounted for 11% of variability in our Holstein population (Figure 2B). Maltecca et al. identified QTL for birth weight on chromosomes 2, 6, and 14 in a crossbred Holstein and Jersey population [5]. There may be other genetic factors that are associated with birth weight on these chromosomes.

We found that the polymorphism in the 5′UTR of *SLC44A5* is correlated with birth weight. The polymorphism is not predicted to directly affect a transcription factor binding site (TRANSFAC 7.0, http://www.gene-regulation.com/pub/datab ases.html), however, the polymorphism might affect interaction with an unknown nuclear protein [25]. Many SNPs associated with a broad range of disease phenotypes alter the RNA structural ensemble [26]. Since the polymorphism in the 5′UTR of *SLC44A5* influences its expression level, the associated genetic variant should harbor the functional effect.

Our results suggest that SLC44A5, which is an intermediate-affinity CTL, increases choline efflux similar to low-affinity OCTs and does not increase choline uptake to as great a degree the high-affinity CHTs. Reports have been inconsistent regarding the function of CTL1, the other member of the intermediate-affinity CTLs. Overexpression of yeast CTL1 does not increase choline uptake in yeast [27], whereas overexpression of mouse CTL1 increases choline uptake in Cos-7 cells [28]. Wong et al. reported that the concentrations of K^+^ and Ca^2+^ affect choline efflux [29]. The intermediate-affinity CTLs may increase and decrease choline uptake under different conditions. Further investigations into the function of SLC44A5 as a choline transporter are required.

The majority of cellular choline is phosphorylated by choline kinase to phosphocholine, which is essential for the formation of membrane phosphatidylcholine in the Kennedy pathway [10]. Our metabolic studies indicated that SLC44A5 did not transport phosphocholine (Figure 4F). Thus SLC44A5 transports choline before entering the Kennedy pathway, suggesting that SLC44A5 might keep the appropriate level of cellular choline.

During pregnancy, fetal plasma choline levels are kept higher than maternal plasma [30], implicating the importance of choline for the developing fetus. Dietary rumen-protected choline improved reproductive performance of Holstein dairy cows [31]. However, supplementing too much choline would increase the birth weight of calves and cause dystocia. Thus SLC44A5 might transport excess choline and keep the appropriate size of fetus.

In conclusion, we found that birth weight in cattle was associated with SLC44A5. SLC44A5 is a choline transporter and the birth weight of cows with the G polymorphism in the 5′ UTR of *SLC44A5* is smaller than that of cows with the A polymorphism. This G polymorphism increases the expression level of SLC44A5. HeLa cells transfected with SLC44A5 decreases proliferation and increases the expression of several markers of apoptosis. Our work identified that SLC44A5 is a critical mediator of birth weight and that SLC44A5 might be a useful target for preventing dystocia.

## Materials and Methods

### Ethics Statement

All animal experimentation was undertaken with the approval of the National Livestock Breeding Center Committee on Animal Research (H21-35).

### QTL Mapping

Genomic DNA was isolated from blood or semen using NA-1000/48S (Kurabo, Tokyo, Japan) or the Easy-DNA kit (Invitrogen, Carlsbad, CA, USA). Fluorescence-labeled (CA)n microsatellite markers were selected based on the Shirakawa-USDA genetic map [32]. The additional markers were developed based on the 7.15X WGS Btau_4.0 assembly (http://www.hgsc.bcm.tmc.edu/projects/bovine/). The primer sequences are available upon request. Genotyping was performed using an ABI 3730 sequencer and GeneMapper (Applied Biosystems, Foster City, CA, USA).

The population structure of our samples was estimated with STRUCTURE [14]. 146 markers were extracted from 1151 markers, with at least a 20-cM interval. We set 100,000 Markov chain Monte Carlo iterations including 10,000 burn-in iterations, and assumed the subpopulation number to be 2. 172 individuals were separated into populations 1 and 2.

The degree of stratification of the samples in this study was examined using the genomic control method [15]. Briefly, λ was the observed median of χ^2^ values of multiple testing divided by the expected median of χ^2^ value (*p* = 0.5) under the null hypothesis. λ indicates degree of inflation of χ^2^ statistic values throughout multiple tests. If there is no stratification, λ is equal to 1. Because the degree of freedom of each test in this study was not always the same, the overall average of λ weighted by the number of tests for each degree of freedom was calculated. χ^2^ values with Yates' correction for continuity were used because the expected value of the cells in the contingency tables was often less than 5.

Fisher's exact test was used for association studies after estimating haplotypes of consecutive marker pairs by expectation-maximization algorithm as described previously [33].

### Genotyping

Each exon from the bovine *ACADM1* and *SLC44A5* genes was sequenced with the primers shown in Tables 1 and 2 following PCR amplification. The National Livestock Breeding Center calculated the dystocia rate for each sire based on their daughters' delivery records.

**Table 1:** A list of *ACADM* primers.

Name	Sequence
5UTRF	Gaccccttgaagcagaaac
5UTRR	CCTAAACAGCGCGATCAT
Exon 1F	CGGAGTAGGCACAAACTGGT
Exon 1R	CCTGATGATTGGGCTTCTTC
Exon 2F	TGACTGATGTTTAAATTCCCAAA
Exon 2R	CCATGTGGCAGCCAATAATA
Exon 3-4F	TTCATTTTCTCAACTCATTGTCCT
Exon 3-4R	TCTTCTTCATGTGCATGCTAGG
Exon 5F	TGCTATGTATTACAATGGGCTCTT
Exon 5R	CAACAGCTCGGTTAGCCAAT
Exon 6F	CTGTGCAGCAAGGCTAGAAA
Exon 6R	GCATGCTTATTCCTTTCTTCTTC
Exon 7F	TTTCCTTTTTCCCATATATTCAAG
Exon 7R	AACAAAGGAAAGGGGAGAGG
Exon 8F	ATTGGGATTGTTGGGAGGAT
Exon 8R	TTGGAGGCCTTAATCACTGT
Exon 9F	ATGCTAGCTCAAAATATGTTTCATAC
Exon 9R	CCAAGCAAACAACATTAAAACAA
Exon 10F	AGGTCAGCTTCTCTTTGCAC
Exon 10R	TGGTTTTACATTTGAACAAAACAAGT
Exon 11F	TGGAGGAAGCAGAGTGTAACTT
Exon 11R	AGGCTCACAGCCACTTATTATG
Exon 12F	GGACTGTCTAAAATACTGAGGACTTAC
Exon 12R	AGAATACAAACAGATACACAAATTGAA

**Table 2:** A list of *SLC44A5* primers.

Name	Sequence
5UTR-Exon 2F	ACAGGAGATGGCCAAGAAGA
5UTR-Exon 2R	TCGCATCTCAGAGTGTGGTC
Exon 3F	TTTTAGGGCAGGGAGAAACA
Exon 3R	GACTATGAAGGGCCGACTGA
Exon 4F	ATTTCATTGGCTTCCATGCT
Exon 4R	TCTGCTTTGGAAGGCTGAGT
Exon 5F	GGTTGCCATTTTCTTCTCCA
Exon 5R	GTGAGGCATACTCCCTCCAG
Exon 6F	AGTGTGTTGCAGTGGTGCAT
Exon 6R	AATCAGCAAGGTGGCCATAC
Exon 7F	TCCTAGAGGAAATGAAGCAGATTT
Exon 7R	GCTAAACCCAGTGAGGCATT
Exon 8F	TCAATTCCTACCACTGTATTTATTTCA
Exon 8R	TTTTGGAGCCTGTCCAGTTA
Exon 9F	TGTGTCGTCACATAGTCTTCG
Exon 9R	CAGGGAGTATGGGCCAGTTA
Exon 10F	GCTGAAGAAAGAGAGGACATCG
Exon 10R	TGGGGTGCATAAAATTGAAA
Exon 11F	CAGCTTCCAGGTCCGTTTTA
Exon 11R	AACTCAGAATGGGCTGGAGA
Exon 12F	ACTGGCCCCCTATGTTTGTT
Exon 12R	TTGACAATGGCAACTGAAAAG
Exon 13F	TCAATTTCATGTAGATGTGTGCTT
Exon 13R	AAAAATGCTTTCTGGTTATTACAATTT
Exon 14F	CTGGCAGAAATCATCACGAA
Exon 14R	TGCGAGTCTCTCCATCAAGG
Exon 15F	TGGTTGTTACCACTGTTCTTTGA
Exon 15R	TGGACCTGAGGTTTAAAGAAGTC
Exon 16F	TTTATTTTCAATCTGCCAGCAA
Exon 16R	CAGTGCACAGGGTCCCTATTA
Exon 17F	AGGGAAGGCATTGTCTTGG
Exon 17R	TTGCCACTTGAATTATAAGCACA
Exon 18F	CCTTTATTCTCATTTTATCTTTGTCCA
Exon 18R	CCAACCCCTGATCCAGTCTA
Exon 19F	CAGTTAAATCAAAAAGCCCATT
Exon 19R	TCGGGTTTGCAAAGAAGTTT
Exon 20F	AGCCCAAAGGTTGGAAAGAT
Exon 20R	CATCCACTGCAATGCTCAAC
Exon 21-22F	TGAATTCATGGATTATTTACTTTCAT
Exon 21-22R	GCAGCCCAAACTTACTATCAGC
Exon 23F	TGGACAAATTTACGACATCTTCA
Exon 23R	TCCACAGCCAGCTAAAGACA
Exon 24F	AATTATTCTGCCGCTGCTTG
Exon 24R	CAAAGTGCTGCAATGGAAAA
Exon 25F	TTACCCTGTGCCATTCTTCC
Exon 25R	CGGGAGTGGGGAAAACTTTA

### Luciferase Assay

Fragments of the 5′ UTR of *SLC44A5* were generated using PCR with the following primers: forward, 5′-CCGAGCTCTTACGCGTGAGATAGGAGCTGTCTGGCT-3′ and reverse, 5′-CTTAGATCGCAGATCTGGTGCCGAGCTCGTTTCCAC-3′. These fragments were then cloned into the pGL3(R2.2)-basic vector (Promega, Madison, MI, USA) and transfected into HeLa cells, which were provided by the RIKEN CELL BANK (Tsukuba, Japan). The transfections were performed with Lipofectamine 2000 (Invitrogen) for 24 h according to standard Invitrogen protocols. The luciferase assays were performed using a SLC44A5-pGL3(R2.2)-basic vector and a pRL-TK vector (Promega) as an internal control (ratio of 10: 1) based on the dual-luciferase reporter assay system (Promega). Each assay was repeated eight times. The measurements were calculated following the subtraction of the background signals for *Renilla* luciferase and were subjected to statistical analysis using the Student's t-test.

### Gel Mobility Shift Assay

Nuclear protein from HeLa cells was extracted using the CelLytic NuClear extraction kit (Sigma Chemical Co., St. Louis, MO, USA). The protein concentration was measured using the Bio-Rad protein assay using bovine serum albumin as the standard (Bio-Rad, Hercules, CA, USA). For the gel mobility shift assay, 5 μg of nuclear protein was used as input for the gel shift assay system (Promega) and was electrophoresed in a 6% retardation gel (Invitrogen). The following probe and competitors were used: 5′-TTGAATTGAATTGAATTGAA-3′ for the A polymorphism and 5′-TTGGATTGGATTGGATTGGA-3′ for the G polymorphism.

### QPCR

RNA was extracted from the bovine brain, jejunum, liver, lung, pancreas, spleen, thymus, fat, and from transfected HeLa cells using TRIzol (Invitrogen). QPCR was conducted with an ABI 7900HT sequence detection system using the comparative Ct method and glyceraldehyde-3-phosphate dehydrogenase (GAPD) as an internal control (Applied Biosystems). Bovine *SLC44A5* was amplified with the following primers: forward, 5′-TGGATCTTACATAATTGCACATGGA-3′; reverse, 5′-TCCAAGAAGCAGATGAAAATTGTT-3′; and probe, 5′-TCTTCAGCGTCTATGCAATGTGTAT-3′. Bovine *GAPD* was amplified with the following primers: forward, 5′-GCCCTCAACGACCACTTTGT-3′; reverse, 5′-CCTGTTGCTGTAGCCAAATTCA-3′; and probe, 5′-AAGCTCATTTCCTGGTACGA-3′. Human *SLC44A5* and *GAPD* were amplified with primers obtained from Applied Biosystems. Each QPCR was subjected to statistical analysis using the Student's t-test.

### SNaPshot and Quantitative Analysis of Allele Ratios

SNaPshot was performed using SNaPshot multiplex kit (Applied Biosystems). The 5′ UTR of *SLC44A5* was amplified by PCR using amplification primers (forward, 5′- CTCTTCCCGACCTGCTGA -3′; and reverse, 5′- CCAAGTGAGTATCTGATCGTTGGT-3′). Amplified PCR products were purified and analyzed using extension primer 5′- GCTCCTTTGGAACCAGGGCTTCTAAAGTTG -3′. Subsequent extension with DNA polymerase added a single fluorescent triphosphate complementary to the nucleotide at the polymorphic site. The extended primers labeled with different fluorescent dyes were analyzed and the peak area ratios were calculated to measure the relative amount of DNA or complementary DNA (cDNA). For each brain tissue, peak area ratios were measured for both DNA and mRNA (cDNA). Assuming that the two alleles were present in equal amounts in genomic DNA, measured DNA and cDNA ratios were normalized to the average of genomic DNA ratios. For cDNA preparations, each mRNA was converted to cDNA in three separate experiments.

### Choline uptake studies

The sequence of the *Bos taurus SLC44A5* gene has been submitted to GenBank with a submission ID of JN590252. Bovine *SLC44A5* coding sequences were derived using reverse-transcription PCR with the following primers: forward, 5′-ATGCGCGGAGACCAACGATCAGATACTCACTT-3′, and reverse, 5′-CTACTGCTTCTTGGTTTCTGCATTTCGCTTGTTCA-3′. The coding sequence was cloned into the pcDNA3.2/V5-DEST vector (Invitrogen) to express SLC44A5 protein, and the resulting plasmid was transfected into HeLa cells. The siRNA against *SLC44A5* and negative control siRNA were obtained from Invitrogen. siRNA transfection was performed with Lipofectamine RNAiMAX (Invitrogen) for 24 h according to the standard Invitrogen protocols.

The choline uptake studies were performed as previously described [34]. Briefly, the culture medium was removed from the 24-well culture plates by aspiration. The cells were then washed twice with uptake buffer consisting of 125 mM NaCl, 4.8 mM KCl, 1.2 mM CaCl_2_, 1.2 mM KH_2_PO_4_, 5.6 mM glucose, 1.2 mM MgSO_4_, and 25 mM HEPES adjusted to pH 7.4 with Tris. [Methyl-^3^H]choline chloride (specific activity: 12.4 nmol/37 MBq/ml) was obtained from PerkinElmer Life Sciences, Inc. (Boston, MA, USA). Choline uptake was initiated by adding 250 μl of uptake buffer containing [^3^H]choline. Following incubation at 37°C in 5% CO_2_ and 95% air, cells were washed twice with ice-cold uptake buffer and lysed in 0.1 M NaOH and 0.1% Triton X-100. Aliquots were then taken for liquid scintillation counting and protein assays. For the efflux measurements, the cells were washed three times with ice-cold uptake buffer following uptake. Two hundred-fifty microliters of uptake buffer was then added, and the cells were incubated at 37°C in an atmosphere of 5% CO_2_ and 95% air. Following incubation, the radioactivity released into the buffer was measured.

### Metabolic studies

The metabolic studies were performed as previously described [35]. Briefly, transfected HeLa cells in 35-mm dishes were incubated with 250 nM [^3^H]choline for 20 min, washed three times, and incubated with 1.25 ml of uptake buffer for 10 min. The collected buffer was completely evaporated under reduced pressure and the residue was dissolved in 90 μl of 50% ethanol. The aliquots were then applied to pre-coated TLC aluminum sheets with a sorbent of silica gel with pore diameters of 60 Å (Merck, Darmstadt, Germany) and chromatographed using the following solvent: methanol/0.5% NaCl/ammonia (100/100/2, v/v/v). The TLC sheets were cut into 5-mm sections, and the radioactivity of the sections was measured. Phosphocholine, glycerophosphocholine, acetylecholine, and choline were used as standards.

### Cell viability assay

Transfected HeLa cells were analyzed with the CellTiter-Glo luminescent cell viability assay (Promega) according to standard Promega protocols. The measurements were subjected to statistical analysis using the Student's t-test. The transfection efficiency was confirmed by co-transfection with the pRL-TK vector (Promega), and measurements were made using the EnduRen live cell substrate (Promega).

### Microarray analysis

RNA extracted from transfected HeLa cell was analyzed with Human genome U133 Plus 2.0 arrays (Affymetrix, Santa Clara, CA, USA). The data from a total of 12 arrays (3 arrays each from NC-, siSLC-, Vector-, and SLC-treated cells) were normalized using the RMA method [36] and were subjected to statistical analysis using the Mann–Whitney U-test. The data from these 12 arrays is available on GEO under the GEO submission ID GSE31434.

## Supporting Information

Figure S1 **The population structure of analyzed samples based on STRUCTURE.** The inferred proportion of ancestry in population 1 of heavy (samples with a birth weight of greater than 51 kg, red) and light (samples with a birth weight of less than 35 kg, blue) were similar.
(TIF)

Figure S2 **The** ***SLC44A5*** **5′ UTR SNP is associated with birth weight.** A. The average birth weight ± SE values for the female calves newly collected. The *p*-value was calculated using the Student's t-test. B. The average birth weight ± SE values for the male calves. The *p*-value was calculated using the Student's t-test.
(TIF)

Table S1 (DOC)
