# Supplementary figures and images for: The Molecular Effects of a Polymorphism in the 5′UTR of Solute Carrier Family 44, Member 5 that Is Associated with Birth Weight in Holsteins
Source: PLoS One. 2012 Jul 18;7(7):e41267. doi: 10.1371/journal.pone.0041267 (PMC3399839; doi:10.1371/journal.pone.0041267)

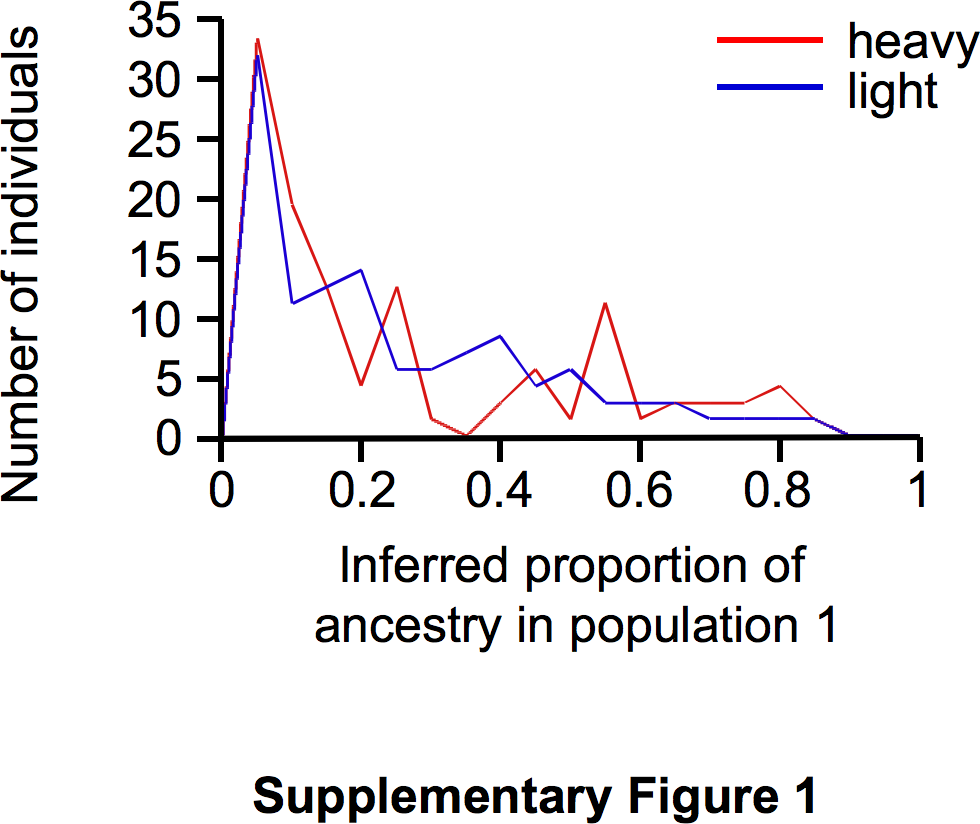

Supplement: Figure S1 — The population structure of analyzed samples based on STRUCTURE. The inferred proportion of ancestry in population 1 of heavy (samples with a birth weight of greater than 51 kg, red) and light (samples with a birth weight of less than 35 kg, blue) were similar. (TIF) [file pone.0041267.s001.tif]

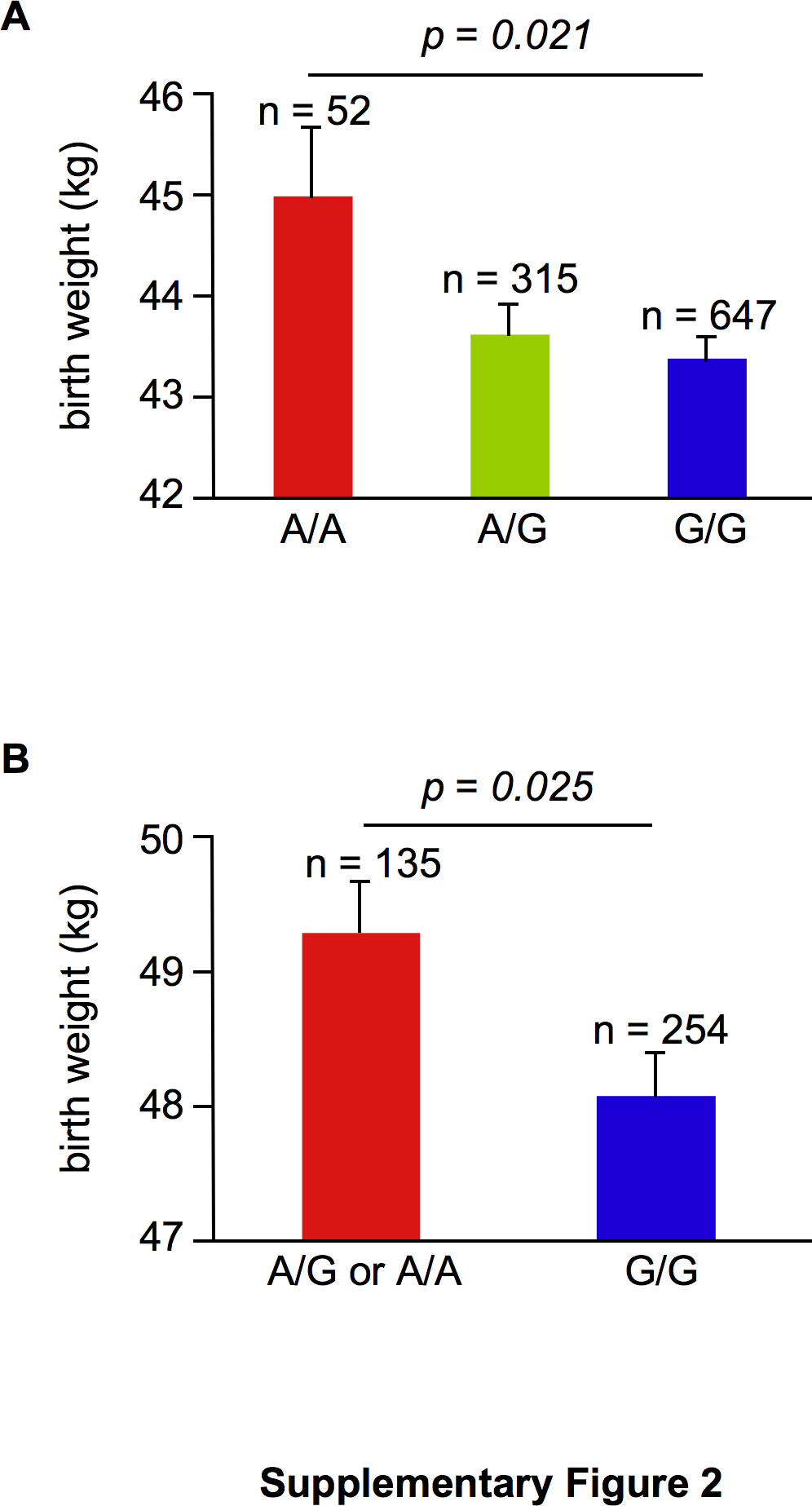

Supplement: Figure S2 — The SLC44A5 5′ UTR SNP is associated with birth weight. A. The average birth weight ± SE values for the female calves newly collected. The p-value was calculated using the Student's t-test. B. The average birth weight ± SE values for the male calves. The p-value was calculated using the Student's t-test. (TIF) [file pone.0041267.s002.tif]
