# Supplementary material for: The Molecular Effects of a Polymorphism in the 5′UTR of Solute Carrier Family 44, Member 5 that Is Associated with Birth Weight in Holsteins
Source: PLoS One. 2012 Jul 18;7(7):e41267. doi: 10.1371/journal.pone.0041267 (PMC3399839; doi:10.1371/journal.pone.0041267)
Supplement: Table S1 — (DOC) [file pone.0041267.s003.doc]

**Supplementary Table 1** A list of probe sets downregulated or upregulated by *SLC44A5*

| Probe Set ID | Gene Symbol | Down or Up |
| --- | --- | --- |
| 1552559_a_at | PFTK2 | Down |
| 1552927_at | MAP3K7IP3 | Down |
| 1553011_at | TAF1L | Down |
| 1553510_s_at | RQCD1 | Down |
| 1553709_a_at | PRPF38A | Down |
| 1554167_a_at | GOLGA7 | Down |
| 1554345_a_at | GIN1 | Down |
| 1554482_a_at | SAR1B | Down |
| 1554547_at | FAM13C | Down |
| 1554768_a_at | MAD2L1 | Down |
| 1554868_s_at | PCNP | Down |
| 1554887_at | --- | Down |
| 1555585_a_at | FAM71B | Down |
| 1556171_a_at | --- | Down |
| 1556285_s_at | PPA2 | Down |
| 1556694_a_at | --- | Down |
| 1557400_at | --- | Down |
| 1558208_at | --- | Down |
| 1559072_a_at | ELFN2 | Down |
| 1560006_a_at | LOC646762 | Down |
| 1560148_at | --- | Down |
| 1560434_x_at | CLTA | Down |
| 1560753_at | --- | Down |
| 1560937_at | RSU1 | Down |
| 1561048_at | RARS2 | Down |
| 1562558_at | LOC440704 | Down |
| 1564466_at | FLJ37644 | Down |
| 1564640_at | MGA | Down |
| 1565131_x_at | MAP3K2 | Down |
| 1565269_s_at | ATF1 | Down |
| 1565876_x_at | --- | Down |
| 1566235_at | DGCR12 | Down |
| 1567377_at | DNAH1 | Down |
| 1568619_s_at | ITPRIPL2 | Down |
| 1569025_s_at | FAM13A | Down |
| 1569253_at | INTS4 | Down |
| 1570255_s_at | ANKRD20A1 /// ANKRD20A2 /// ANKRD20A3 /// ANKRD20A4 /// ANKRD20B /// LOC375010 | Down |
| 200030_s_at | SLC25A3 | Down |
| 200070_at | C2orf24 | Down |
| 200079_s_at | KARS | Down |
| 200659_s_at | PHB | Down |
| 200670_at | XBP1 | Down |
| 200681_at | GLO1 | Down |
| 200764_s_at | CTNNA1 | Down |
| 200811_at | CIRBP | Down |
| 200833_s_at | hCG_1757335 /// RAP1B | Down |
| 200844_s_at | PRDX6 | Down |
| 200883_at | UQCRC2 | Down |
| 200943_at | HMGN1 | Down |
| 200980_s_at | PDHA1 | Down |
| 200992_at | IPO7 | Down |
| 200997_at | RBM4 | Down |
| 201139_s_at | SSB | Down |
| 201209_at | HDAC1 | Down |
| 201223_s_at | RAD23B | Down |
| 201277_s_at | HNRNPAB | Down |
| 201375_s_at | PPP2CB | Down |
| 201404_x_at | PSMB2 | Down |
| 201435_s_at | EIF4E | Down |
| 201445_at | CNN3 | Down |
| 201452_at | RHEB | Down |
| 201523_x_at | UBE2N | Down |
| 201524_x_at | UBE2N | Down |
| 201570_at | SAMM50 | Down |
| 201595_s_at | ZC3H15 | Down |
| 201614_s_at | RUVBL1 | Down |
| 201633_s_at | CYB5B | Down |
| 201695_s_at | NP | Down |
| 201816_s_at | GBAS | Down |
| 201842_s_at | EFEMP1 | Down |
| 202101_s_at | RALB | Down |
| 202119_s_at | CPNE3 | Down |
| 202142_at | COPS8 | Down |
| 202143_s_at | COPS8 | Down |
| 202147_s_at | IFRD1 | Down |
| 202472_at | MPI | Down |
| 202482_x_at | RANBP1 | Down |
| 202483_s_at | RANBP1 | Down |
| 202484_s_at | MBD2 | Down |
| 202511_s_at | ATG5 | Down |
| 202542_s_at | AIMP1 | Down |
| 202680_at | GTF2E2 | Down |
| 202706_s_at | UMPS | Down |
| 202710_at | BET1 | Down |
| 202845_s_at | RALBP1 | Down |
| 202966_at | CAPN6 | Down |
| 203184_at | FBN2 | Down |
| 203210_s_at | RFC5 | Down |
| 203394_s_at | HES1 | Down |
| 203474_at | IQGAP2 | Down |
| 203527_s_at | APC | Down |
| 203586_s_at | ARL4D | Down |
| 203622_s_at | PNO1 | Down |
| 203673_at | TG | Down |
| 203680_at | PRKAR2B | Down |
| 203700_s_at | DIO2 | Down |
| 203711_s_at | HIBCH | Down |
| 203721_s_at | UTP18 | Down |
| 203735_x_at | PPFIBP1 | Down |
| 203820_s_at | IGF2BP3 | Down |
| 203992_s_at | KDM6A | Down |
| 204372_s_at | KHSRP | Down |
| 204389_at | MAOA | Down |
| 204784_s_at | MLF1 | Down |
| 204992_s_at | PFN2 | Down |
| 205088_at | MAMLD1 | Down |
| 205246_at | PEX13 | Down |
| 205352_at | SERPINI1 | Down |
| 205412_at | ACAT1 | Down |
| 205512_s_at | AIFM1 | Down |
| 205565_s_at | FXN | Down |
| 205621_at | ALKBH1 | Down |
| 205741_s_at | DTNA | Down |
| 205805_s_at | ROR1 | Down |
| 205892_s_at | FABP1 | Down |
| 206008_at | TGM1 | Down |
| 206158_s_at | CNBP | Down |
| 206554_x_at | SETMAR | Down |
| 206559_x_at | EEF1A1 | Down |
| 207120_at | ZNF667 | Down |
| 207690_at | ALX3 | Down |
| 208091_s_at | VOPP1 | Down |
| 208373_s_at | P2RY6 | Down |
| 208398_s_at | TBPL1 | Down |
| 208447_s_at | PRPS1 | Down |
| 208456_s_at | RRAS2 | Down |
| 208692_at | RPS3 | Down |
| 208724_s_at | RAB1A | Down |
| 208761_s_at | SUMO1 | Down |
| 208781_x_at | SNX3 | Down |
| 208787_at | MRPL3 | Down |
| 208822_s_at | DAP3 | Down |
| 208877_at | PAK2 | Down |
| 208905_at | CYCS | Down |
| 208920_at | SRI | Down |
| 208951_at | ALDH7A1 | Down |
| 208967_s_at | AK2 | Down |
| 209039_x_at | EHD1 | Down |
| 209092_s_at | GLOD4 | Down |
| 209139_s_at | PRKRA | Down |
| 209153_s_at | TCF3 | Down |
| 209273_s_at | ISCA1 | Down |
| 209297_at | ITSN1 | Down |
| 209337_at | PSIP1 | Down |
| 209406_at | BAG2 | Down |
| 209959_at | NR4A3 | Down |
| 210137_s_at | DCTD | Down |
| 210372_s_at | TPD52L1 | Down |
| 210567_s_at | SKP2 | Down |
| 210639_s_at | ATG5 | Down |
| 210648_x_at | SNX3 | Down |
| 210653_s_at | BCKDHB | Down |
| 210825_s_at | PEBP1 | Down |
| 210993_s_at | SMAD1 | Down |
| 211078_s_at | STK3 | Down |
| 211133_x_at | LILRA6 /// LILRB3 | Down |
| 211267_at | HESX1 | Down |
| 211318_s_at | RAE1 | Down |
| 211518_s_at | BMP4 | Down |
| 211523_at | GNRHR | Down |
| 211563_s_at | C19orf2 | Down |
| 211569_s_at | HADH | Down |
| 211754_s_at | SLC25A17 | Down |
| 211912_at | MERTK | Down |
| 211933_s_at | HNRNPA3 /// HNRNPA3P1 | Down |
| 212018_s_at | RSL1D1 | Down |
| 212038_s_at | VDAC1 | Down |
| 212057_at | KIAA0182 | Down |
| 212175_s_at | AK2 | Down |
| 212234_at | ASXL1 | Down |
| 212282_at | TMEM97 | Down |
| 212296_at | PSMD14 | Down |
| 212385_at | TCF4 | Down |
| 212440_at | SNRNP27 | Down |
| 212507_at | TMEM131 | Down |
| 212510_at | GPD1L | Down |
| 212656_at | TSFM | Down |
| 212773_s_at | TOMM20 | Down |
| 212815_at | ASCC3 | Down |
| 212993_at | NACC2 | Down |
| 213098_at | RQCD1 | Down |
| 213144_at | GOSR2 | Down |
| 213237_at | C16orf88 | Down |
| 213302_at | PFAS | Down |
| 213310_at | EIF2C2 | Down |
| 213849_s_at | PPP2R2B | Down |
| 214167_s_at | RPLP0 /// RPLP0P6 | Down |
| 214358_at | ACACA | Down |
| 214442_s_at | PIAS2 | Down |
| 214553_s_at | ARPP19 | Down |
| 214590_s_at | UBE2D1 | Down |
| 214743_at | CUX1 | Down |
| 214956_at | AAK1 | Down |
| 215234_at | --- | Down |
| 215517_at | PYGO1 | Down |
| 215643_at | --- | Down |
| 215849_x_at | TTC18 | Down |
| 216127_at | PDIA2 | Down |
| 217364_x_at | --- | Down |
| 217748_at | ADIPOR1 | Down |
| 217808_s_at | MAPKAP1 | Down |
| 217809_at | BZW2 | Down |
| 217850_at | GNL3 | Down |
| 217860_at | LOC732160 /// NDUFA10 | Down |
| 217932_at | MRPS7 | Down |
| 217945_at | BTBD1 | Down |
| 217968_at | TSSC1 | Down |
| 218025_s_at | PECI | Down |
| 218027_at | MRPL15 | Down |
| 218165_at | MEAF6 | Down |
| 218271_s_at | PARL | Down |
| 218616_at | INTS12 | Down |
| 218711_s_at | SDPR | Down |
| 218868_at | ACTR3B | Down |
| 218897_at | TMEM177 | Down |
| 218998_at | C9orf6 | Down |
| 219015_s_at | ALG13 | Down |
| 219185_at | SIRT5 | Down |
| 219478_at | WFDC1 | Down |
| 219686_at | STK32B | Down |
| 219905_at | ERMAP | Down |
| 219940_s_at | PCID2 | Down |
| 220143_x_at | LUC7L | Down |
| 220155_s_at | BRD9 | Down |
| 220175_s_at | CBWD1 /// CBWD2 /// CBWD3 /// CBWD5 /// CBWD6 /// CBWD7 | Down |
| 220212_s_at | THADA | Down |
| 220858_at | SORBS2 | Down |
| 220865_s_at | PDSS1 | Down |
| 220943_s_at | C2orf56 | Down |
| 221021_s_at | CTNNBL1 | Down |
| 221072_at | C9orf31 | Down |
| 221094_s_at | ELP3 | Down |
| 221381_s_at | MORF4 /// MORF4L1 | Down |
| 221541_at | CRISPLD2 | Down |
| 221580_s_at | TAF1D | Down |
| 221598_s_at | MED27 | Down |
| 221964_at | TULP3 | Down |
| 222032_s_at | USP7 | Down |
| 222441_x_at | SLMO2 | Down |
| 222494_at | FOXN3 | Down |
| 222578_s_at | UBA5 | Down |
| 222617_s_at | C10orf84 | Down |
| 222623_s_at | ZNF639 | Down |
| 222666_s_at | RCL1 | Down |
| 222725_s_at | PALMD | Down |
| 222755_s_at | CHD7 | Down |
| 222837_s_at | NARG1 | Down |
| 222877_at | --- | Down |
| 222930_s_at | AGMAT | Down |
| 222983_s_at | PAIP2 | Down |
| 222984_at | PAIP2 | Down |
| 223004_s_at | C3orf1 | Down |
| 223034_s_at | C1orf43 | Down |
| 223035_s_at | FARSB | Down |
| 223124_s_at | C1orf128 | Down |
| 223125_s_at | C1orf21 | Down |
| 223158_s_at | NEK6 | Down |
| 223161_at | KIAA1147 | Down |
| 223180_s_at | C18orf55 | Down |
| 223221_at | SCO1 | Down |
| 223267_at | RG9MTD1 | Down |
| 223366_at | --- | Down |
| 223397_s_at | NIP7 | Down |
| 223472_at | WHSC1 | Down |
| 223546_x_at | LUC7L | Down |
| 223559_s_at | C9orf80 | Down |
| 223996_s_at | MRPL30 | Down |
| 224143_at | LOC100101118 /// TTTY8 | Down |
| 224153_s_at | C14orf167 | Down |
| 224232_s_at | PRELID1 | Down |
| 224247_s_at | MRPS10 | Down |
| 224280_s_at | FAM54B | Down |
| 224561_s_at | MORF4L1 | Down |
| 224623_at | LOC728554 /// THOC3 | Down |
| 224713_at | MKI67IP | Down |
| 224740_at | C5orf43 | Down |
| 224769_at | --- | Down |
| 224787_s_at | RAB18 | Down |
| 224811_at | --- | Down |
| 224879_at | C9orf123 | Down |
| 224894_at | YAP1 | Down |
| 224899_s_at | MAGT1 | Down |
| 224937_at | PTGFRN | Down |
| 225120_at | PURB | Down |
| 225126_at | MRRF | Down |
| 225149_at | PCID2 | Down |
| 225160_x_at | MDM2 | Down |
| 225202_at | RHOBTB3 | Down |
| 225209_s_at | UBE2J2 | Down |
| 225380_at | SGK493 | Down |
| 225424_at | GPAM | Down |
| 225444_at | UBN2 | Down |
| 225504_at | --- | Down |
| 225549_at | DDX6 | Down |
| 225575_at | LIFR | Down |
| 225664_at | COL12A1 | Down |
| 225670_at | FAM173B | Down |
| 225710_at | GNB4 | Down |
| 225762_x_at | LOC284801 | Down |
| 225767_at | --- | Down |
| 225836_s_at | C12orf32 | Down |
| 226116_at | --- | Down |
| 226127_at | ALKBH3 | Down |
| 226492_at | SEMA6D | Down |
| 226547_at | MYST3 | Down |
| 226616_s_at | NDUFV3 | Down |
| 226810_at | OGFRL1 | Down |
| 226934_at | CPSF6 | Down |
| 227115_at | --- | Down |
| 227173_s_at | BACH2 | Down |
| 227947_at | PHACTR2 | Down |
| 227966_s_at | CCDC74A /// CCDC74B | Down |
| 227996_at | FARP1 | Down |
| 228184_at | DISP1 | Down |
| 228501_at | GALNTL2 | Down |
| 228555_at | CAMK2D | Down |
| 228564_at | LOC375295 | Down |
| 229144_at | RP1-21O18.1 | Down |
| 229236_s_at | SFXN4 | Down |
| 229296_at | --- | Down |
| 229404_at | TWIST2 | Down |
| 229513_at | STRBP | Down |
| 229595_at | CHCHD4 | Down |
| 229604_at | CMAH | Down |
| 229612_at | --- | Down |
| 229676_at | MTPAP | Down |
| 229786_at | --- | Down |
| 229845_at | MAPKAP1 | Down |
| 229846_s_at | MAPKAP1 | Down |
| 229980_s_at | SNX5 | Down |
| 229994_at | NFIA | Down |
| 230995_at | CMBL | Down |
| 231240_at | DIO2 | Down |
| 231791_at | ASAH2B | Down |
| 231851_at | RAVER2 | Down |
| 231983_at | C1orf69 | Down |
| 232560_at | UROS | Down |
| 232902_s_at | RARS2 | Down |
| 233062_at | --- | Down |
| 233124_s_at | ECHDC1 | Down |
| 233458_at | POLR3E | Down |
| 233496_s_at | CFL2 | Down |
| 233836_at | TNRC6A | Down |
| 233999_s_at | TTC26 | Down |
| 234067_at | --- | Down |
| 234926_s_at | C20orf43 | Down |
| 234990_at | CBX5 | Down |
| 235173_at | hCG_1806964 | Down |
| 235260_s_at | PACRGL | Down |
| 235371_at | GLT8D4 | Down |
| 235501_at | --- | Down |
| 235524_at | GARNL1 | Down |
| 236202_at | --- | Down |
| 236277_at | --- | Down |
| 236535_at | SMC6 | Down |
| 236537_at | --- | Down |
| 236571_at | --- | Down |
| 236900_x_at | LOC126661 | Down |
| 237233_at | --- | Down |
| 237575_at | --- | Down |
| 237679_at | TRIM66 | Down |
| 237752_at | --- | Down |
| 238093_at | LOC100129722 | Down |
| 238300_s_at | DAZAP2 | Down |
| 238404_x_at | SEZ6L2 | Down |
| 238445_x_at | MGAT5B | Down |
| 238761_at | --- | Down |
| 238834_at | MYLK3 | Down |
| 239225_at | --- | Down |
| 240011_at | TTBK2 | Down |
| 240040_at | PSTK | Down |
| 241130_at | --- | Down |
| 241266_at | --- | Down |
| 241531_at | --- | Down |
| 241838_at | --- | Down |
| 241965_at | --- | Down |
| 241990_at | RHOV | Down |
| 242426_at | NRG4 | Down |
| 242474_s_at | VMA21 | Down |
| 242542_at | --- | Down |
| 242735_x_at | ELF2 | Down |
| 243010_at | MSI2 | Down |
| 243023_at | --- | Down |
| 243184_at | --- | Down |
| 243229_at | --- | Down |
| 243495_s_at | --- | Down |
| 244253_at | --- | Down |
| 244625_at | --- | Down |
| 49452_at | ACACB | Down |
| 1405_i_at | CCL5 | Up |
| 1552327_at | ARMCX4 | Up |
| 1552685_a_at | GRHL1 | Up |
| 1553023_a_at | NOX5 | Up |
| 1553099_at | TIGD1 | Up |
| 1553299_at | DUSP5P | Up |
| 1553407_at | MACF1 | Up |
| 1553423_a_at | SLFN13 | Up |
| 1553677_a_at | TIPRL | Up |
| 1554015_a_at | CHD2 | Up |
| 1554018_at | GPNMB | Up |
| 1554195_a_at | C5orf46 | Up |
| 1554250_s_at | TRIM73 | Up |
| 1554355_a_at | SIAE | Up |
| 1554721_a_at | TAF2 | Up |
| 1554997_a_at | PTGS2 | Up |
| 1555074_a_at | KCNH5 | Up |
| 1555301_a_at | DIP2A | Up |
| 1555363_s_at | LOC284440 | Up |
| 1555759_a_at | CCL5 | Up |
| 1555845_at | --- | Up |
| 1555854_at | --- | Up |
| 1555976_s_at | --- | Up |
| 1556151_at | ITFG1 | Up |
| 1556242_a_at | --- | Up |
| 1556301_at | --- | Up |
| 1556420_s_at | YPEL2 | Up |
| 1556700_a_at | --- | Up |
| 1556773_at | --- | Up |
| 1556942_at | --- | Up |
| 1557064_s_at | HGSNAT | Up |
| 1557078_at | SLFN5 | Up |
| 1557137_at | TMEM17 | Up |
| 1557236_at | APOL6 | Up |
| 1557292_a_at | MCOLN3 | Up |
| 1557385_at | FAM161A | Up |
| 1557431_at | --- | Up |
| 1558143_a_at | BCL2L11 | Up |
| 1558310_s_at | hCG_2039148 | Up |
| 1558517_s_at | LRRC8C | Up |
| 1559114_a_at | CXCR7 | Up |
| 1559439_s_at | C21orf58 | Up |
| 1559617_at | --- | Up |
| 1560019_at | MGC11082 | Up |
| 1560260_at | LOC285593 | Up |
| 1560522_at | LOC201477 | Up |
| 1560821_at | ARHGAP22 | Up |
| 1560957_at | --- | Up |
| 1560974_s_at | NOS1 | Up |
| 1561144_at | --- | Up |
| 1561197_at | LOC100294357 /// LOC442028 | Up |
| 1561451_a_at | ADAM29 | Up |
| 1562062_at | KIAA1245 /// NBPF1 /// NBPF10 /// NBPF11 /// NBPF12 /// NBPF20 /// NBPF3 /// NBPF8 /// RP11-94I2.2 | Up |
| 1562084_at | --- | Up |
| 1562772_a_at | DAND5 | Up |
| 1564130_x_at | --- | Up |
| 1564190_x_at | ZNF519 | Up |
| 1564373_a_at | LOC283887 | Up |
| 1565320_at | RBMY3AP | Up |
| 1566256_s_at | --- | Up |
| 1566540_at | --- | Up |
| 1567107_s_at | TPM4 | Up |
| 1567224_at | HMGA2 | Up |
| 1567304_at | --- | Up |
| 1568604_a_at | CADPS | Up |
| 1568612_at | GABRG2 | Up |
| 1569307_s_at | --- | Up |
| 1569674_at | BHLHB9 | Up |
| 1570444_at | LOC643201 | Up |
| 200635_s_at | PTPRF | Up |
| 200677_at | PTTG1IP | Up |
| 200745_s_at | GNB1 | Up |
| 200815_s_at | PAFAH1B1 | Up |
| 200887_s_at | STAT1 | Up |
| 200905_x_at | HLA-E | Up |
| 200956_s_at | SSRP1 | Up |
| 201005_at | CD9 | Up |
| 201061_s_at | STOM | Up |
| 201065_s_at | GTF2I /// GTF2IP1 /// LOC100093631 | Up |
| 201078_at | TM9SF2 | Up |
| 201116_s_at | CPE | Up |
| 201170_s_at | BHLHE40 | Up |
| 201300_s_at | PRNP | Up |
| 201449_at | TIA1 | Up |
| 201626_at | INSIG1 | Up |
| 201631_s_at | IER3 | Up |
| 201661_s_at | ACSL3 | Up |
| 201723_s_at | GALNT1 | Up |
| 201824_at | RNF14 | Up |
| 201847_at | LIPA | Up |
| 201897_s_at | CKS1B | Up |
| 201925_s_at | CD55 | Up |
| 201952_at | ALCAM | Up |
| 201991_s_at | KIF5B | Up |
| 202068_s_at | LDLR | Up |
| 202086_at | MX1 | Up |
| 202087_s_at | CTSL1 | Up |
| 202211_at | ARFGAP3 | Up |
| 202266_at | TTRAP | Up |
| 202307_s_at | TAP1 | Up |
| 202357_s_at | C2 /// CFB | Up |
| 202411_at | IFI27 | Up |
| 202430_s_at | PLSCR1 | Up |
| 202594_at | LEPROTL1 | Up |
| 202609_at | EPS8 | Up |
| 202859_x_at | IL8 | Up |
| 202863_at | SP100 | Up |
| 202864_s_at | SP100 | Up |
| 202869_at | OAS1 | Up |
| 202909_at | EPM2AIP1 | Up |
| 202952_s_at | ADAM12 | Up |
| 203058_s_at | PAPSS2 | Up |
| 203117_s_at | PAN2 | Up |
| 203123_s_at | SLC11A2 | Up |
| 203124_s_at | SLC11A2 | Up |
| 203265_s_at | MAP2K4 | Up |
| 203413_at | NELL2 | Up |
| 203491_s_at | CEP57 | Up |
| 203724_s_at | RUFY3 | Up |
| 203750_s_at | RARA | Up |
| 203885_at | RAB21 | Up |
| 203955_at | KIAA0649 | Up |
| 204279_at | PSMB9 | Up |
| 204415_at | IFI6 | Up |
| 204417_at | GALC | Up |
| 204465_s_at | INA | Up |
| 204470_at | CXCL1 | Up |
| 204477_at | RABIF | Up |
| 204502_at | SAMHD1 | Up |
| 204508_s_at | CA12 | Up |
| 204544_at | HPS5 | Up |
| 204573_at | CROT | Up |
| 204602_at | DKK1 | Up |
| 204655_at | CCL5 | Up |
| 204698_at | ISG20 | Up |
| 204715_at | PANX1 | Up |
| 204748_at | PTGS2 | Up |
| 204821_at | BTN3A3 | Up |
| 204846_at | CP | Up |
| 204948_s_at | FST | Up |
| 204974_at | RAB3A | Up |
| 205003_at | DOCK4 | Up |
| 205120_s_at | SGCB | Up |
| 205169_at | RBBP5 | Up |
| 205173_x_at | CD58 | Up |
| 205184_at | GNG4 | Up |
| 205198_s_at | ATP7A | Up |
| 205239_at | AREG | Up |
| 205310_at | FBXO46 | Up |
| 205483_s_at | ISG15 | Up |
| 205513_at | TCN1 | Up |
| 205619_s_at | MEOX1 | Up |
| 205809_s_at | WASL | Up |
| 205827_at | CCK | Up |
| 205845_at | CACNA1H | Up |
| 205869_at | PRSS1 | Up |
| 205894_at | ARSE | Up |
| 206026_s_at | TNFAIP6 | Up |
| 206087_x_at | HFE | Up |
| 206100_at | CPM | Up |
| 206173_x_at | GABPB1 | Up |
| 206180_x_at | ZNF747 | Up |
| 206254_at | EGF | Up |
| 206332_s_at | IFI16 | Up |
| 206504_at | CYP24A1 | Up |
| 206526_at | RIBC2 | Up |
| 206540_at | GLB1L | Up |
| 206549_at | INSL4 | Up |
| 206567_s_at | PHF20 | Up |
| 206602_s_at | HOXD3 | Up |
| 206671_at | SAG | Up |
| 206698_at | XK | Up |
| 206842_at | KCND1 | Up |
| 207018_s_at | RAB27B | Up |
| 207631_at | NBR2 | Up |
| 207648_at | DRP2 | Up |
| 207850_at | CXCL3 | Up |
| 207900_at | CCL17 | Up |
| 208012_x_at | SP110 | Up |
| 208107_s_at | LOC81691 | Up |
| 208184_s_at | TRAPPC10 | Up |
| 208206_s_at | RASGRP2 | Up |
| 208367_x_at | CYP3A4 | Up |
| 208433_s_at | LRP8 | Up |
| 208436_s_at | IRF7 | Up |
| 208458_at | SCNN1D | Up |
| 208498_s_at | AMY1A /// AMY1B /// AMY1C /// AMY2A /// AMY2B | Up |
| 208685_x_at | BRD2 | Up |
| 208710_s_at | AP3D1 | Up |
| 208737_at | ATP6V1G1 | Up |
| 208790_s_at | PTRF | Up |
| 208939_at | SEPHS1 | Up |
| 208949_s_at | LGALS3 | Up |
| 208965_s_at | IFI16 | Up |
| 208966_x_at | IFI16 | Up |
| 209019_s_at | PINK1 | Up |
| 209344_at | TPM4 | Up |
| 209351_at | KRT14 | Up |
| 209408_at | KIF2C | Up |
| 209510_at | RNF139 | Up |
| 209771_x_at | CD24 | Up |
| 209774_x_at | CXCL2 | Up |
| 209894_at | LEPR | Up |
| 209917_s_at | TP53TG1 | Up |
| 209921_at | SLC7A11 | Up |
| 209934_s_at | ATP2C1 | Up |
| 209945_s_at | GSK3B | Up |
| 210102_at | VWA5A | Up |
| 210140_at | CST7 | Up |
| 210214_s_at | BMPR2 | Up |
| 210230_at | --- | Up |
| 210233_at | IL1RAP | Up |
| 210355_at | PTHLH | Up |
| 210510_s_at | NRP1 | Up |
| 210524_x_at | --- | Up |
| 210592_s_at | SAT1 | Up |
| 210654_at | TNFRSF10D | Up |
| 210710_at | AGGF1 | Up |
| 210735_s_at | CA12 | Up |
| 210895_s_at | CD86 | Up |
| 211416_x_at | GGTLC1 | Up |
| 211499_s_at | MAPK11 | Up |
| 211519_s_at | KIF2C | Up |
| 211744_s_at | CD58 | Up |
| 211769_x_at | SERINC3 | Up |
| 211851_x_at | BRCA1 | Up |
| 211965_at | ZFP36L1 | Up |
| 212043_at | TGOLN2 | Up |
| 212067_s_at | C1R | Up |
| 212111_at | STX12 | Up |
| 212185_x_at | MT2A | Up |
| 212221_x_at | IDS | Up |
| 212223_at | IDS | Up |
| 212313_at | CHMP7 | Up |
| 212337_at | TUG1 | Up |
| 212353_at | SULF1 | Up |
| 212447_at | KBTBD2 | Up |
| 212498_at | --- | Up |
| 212511_at | PICALM | Up |
| 212613_at | BTN3A2 | Up |
| 212725_s_at | TUG1 | Up |
| 212794_s_at | KIAA1033 | Up |
| 212795_at | KIAA1033 | Up |
| 212928_at | TSPYL4 | Up |
| 212934_at | UBXN2B | Up |
| 212959_s_at | GNPTAB | Up |
| 213042_s_at | ATP2A3 | Up |
| 213361_at | TDRD7 | Up |
| 213393_at | MFSD9 | Up |
| 213396_s_at | --- | Up |
| 213434_at | STX2 | Up |
| 213626_at | CBR4 | Up |
| 213742_at | SFRS11 | Up |
| 213763_at | HIPK2 | Up |
| 213857_s_at | CD47 | Up |
| 214022_s_at | IFITM1 | Up |
| 214059_at | IFI44 | Up |
| 214075_at | NENF | Up |
| 214163_at | HSPB11 | Up |
| 214164_x_at | CA12 | Up |
| 214211_at | FTH1 | Up |
| 214232_at | --- | Up |
| 214334_x_at | DAZAP2 | Up |
| 214469_at | HIST1H2AE | Up |
| 214577_at | MAP1B | Up |
| 214602_at | COL4A4 | Up |
| 214749_s_at | ARMCX6 /// LOC653354 | Up |
| 214773_x_at | TIPRL | Up |
| 214866_at | PLAUR | Up |
| 214911_s_at | BRD2 | Up |
| 215081_at | KIAA1024 | Up |
| 215084_s_at | LRRC42 | Up |
| 215215_s_at | LOC81691 | Up |
| 215493_x_at | BTN2A1 | Up |
| 215819_s_at | RHCE /// RHD | Up |
| 215867_x_at | CA12 | Up |
| 216048_s_at | RHOBTB3 | Up |
| 216224_s_at | HDAC6 | Up |
| 216379_x_at | CD24 | Up |
| 216783_at | --- | Up |
| 216885_s_at | DCAF8 | Up |
| 217526_at | NFATC2IP | Up |
| 217527_s_at | NFATC2IP | Up |
| 217590_s_at | TRPA1 | Up |
| 217609_at | LRRC23 | Up |
| 217678_at | SLC7A11 | Up |
| 217893_s_at | AKIRIN1 | Up |
| 217947_at | CMTM6 | Up |
| 218130_at | C17orf62 | Up |
| 218205_s_at | MKNK2 | Up |
| 218285_s_at | BDH2 | Up |
| 218498_s_at | ERO1L | Up |
| 218543_s_at | PARP12 | Up |
| 218564_at | RFWD3 | Up |
| 218645_at | ZNF277 | Up |
| 218776_s_at | TMEM62 | Up |
| 218932_at | ZNHIT6 | Up |
| 218943_s_at | DDX58 | Up |
| 219024_at | PLEKHA1 | Up |
| 219053_s_at | VPS37C | Up |
| 219069_at | ANKRD49 | Up |
| 219077_s_at | WWOX | Up |
| 219209_at | IFIH1 | Up |
| 219211_at | USP18 | Up |
| 219225_at | PGBD5 | Up |
| 219235_s_at | PHACTR4 | Up |
| 219238_at | PIGV | Up |
| 219247_s_at | ZDHHC14 | Up |
| 219481_at | TTC13 | Up |
| 219663_s_at | TMEM121 | Up |
| 219786_at | MTL5 | Up |
| 219802_at | PYROXD1 | Up |
| 219863_at | HERC5 | Up |
| 219926_at | POPDC3 | Up |
| 219970_at | GIPC2 | Up |
| 219999_at | MAN2A2 | Up |
| 220255_at | FANCE | Up |
| 220295_x_at | DEPDC1 | Up |
| 220305_at | MAVS | Up |
| 220334_at | RGS17 | Up |
| 220370_s_at | USP36 | Up |
| 220399_at | NCRNA00115 | Up |
| 220658_s_at | ARNTL2 | Up |
| 221041_s_at | SLC17A5 | Up |
| 221151_at | PRDM9 | Up |
| 221211_s_at | C21orf7 | Up |
| 221218_s_at | TPK1 | Up |
| 221542_s_at | ERLIN2 | Up |
| 221543_s_at | ERLIN2 | Up |
| 221561_at | SOAT1 | Up |
| 221841_s_at | KLF4 | Up |
| 221905_at | CYLD | Up |
| 221924_at | ZMIZ2 | Up |
| 222111_at | --- | Up |
| 222343_at | BCL2L11 | Up |
| 222392_x_at | PERP | Up |
| 222395_s_at | UBE2Z | Up |
| 222401_s_at | TMEM50A | Up |
| 222407_s_at | ZFP106 | Up |
| 222586_s_at | OSBPL11 | Up |
| 222627_at | VPS54 | Up |
| 222753_s_at | SPCS3 | Up |
| 222775_s_at | MRPL35 | Up |
| 222793_at | DDX58 | Up |
| 222896_at | TMEM38A | Up |
| 223135_s_at | BBX | Up |
| 223220_s_at | PARP9 | Up |
| 223441_at | SLC17A5 | Up |
| 223463_at | RAB23 | Up |
| 223467_at | RASD1 | Up |
| 223547_at | JKAMP | Up |
| 223584_s_at | KBTBD2 | Up |
| 223586_at | ARNTL2 | Up |
| 223715_at | BRSK2 | Up |
| 223824_at | RNLS | Up |
| 223924_at | TTC25 | Up |
| 223981_at | NIN | Up |
| 223984_s_at | NUPL1 | Up |
| 224204_x_at | ARNTL2 | Up |
| 224301_x_at | H2AFJ | Up |
| 224335_s_at | BACE1 | Up |
| 224442_at | PHF6 | Up |
| 224454_at | ETNK1 | Up |
| 224599_at | CGGBP1 | Up |
| 224618_at | ROD1 | Up |
| 224820_at | FAM36A | Up |
| 224848_at | CDK6 | Up |
| 224918_x_at | MGST1 | Up |
| 224942_at | PAPPA | Up |
| 224989_at | --- | Up |
| 225032_at | FNDC3B | Up |
| 225034_at | ST3GAL1 | Up |
| 225108_at | AGPS | Up |
| 225113_at | AGPS | Up |
| 225169_at | INTS4 | Up |
| 225227_at | --- | Up |
| 225306_s_at | SLC25A29 | Up |
| 225308_s_at | TANC1 | Up |
| 225339_at | SPAG9 | Up |
| 225354_s_at | SH3BGRL2 | Up |
| 225415_at | DTX3L | Up |
| 225464_at | FRMD6 | Up |
| 225508_at | KIAA1468 | Up |
| 225516_at | SLC7A2 | Up |
| 225537_at | TRAPPC6B | Up |
| 225574_at | RWDD4A | Up |
| 225612_s_at | B3GNT5 | Up |
| 225657_at | LOC152217 | Up |
| 225661_at | IFNAR1 | Up |
| 225697_at | CRKRS | Up |
| 225711_at | ARL6IP6 | Up |
| 225915_at | CAB39L | Up |
| 225949_at | NRBP2 | Up |
| 226016_at | CD47 | Up |
| 226084_at | MAP1B | Up |
| 226104_at | RNF170 | Up |
| 226125_at | --- | Up |
| 226140_s_at | OTUD1 | Up |
| 226161_at | SLC30A6 | Up |
| 226189_at | ITGB8 | Up |
| 226230_at | SMEK2 | Up |
| 226247_at | PLEKHA1 | Up |
| 226368_at | CHST11 | Up |
| 226493_at | KCTD18 | Up |
| 226552_at | IER5L | Up |
| 226567_at | USP14 | Up |
| 226603_at | SAMD9L | Up |
| 226621_at | --- | Up |
| 226725_at | --- | Up |
| 226730_s_at | USP37 | Up |
| 226757_at | IFIT2 | Up |
| 226760_at | MBTPS2 | Up |
| 226792_s_at | KIFC2 | Up |
| 226802_s_at | LOC96610 | Up |
| 226847_at | FST | Up |
| 226869_at | MEGF6 | Up |
| 227044_at | --- | Up |
| 227065_at | RNF216 | Up |
| 227099_s_at | LOC387763 | Up |
| 227100_at | B3GALTL | Up |
| 227144_at | C22orf9 | Up |
| 227259_at | CD47 | Up |
| 227264_at | --- | Up |
| 227305_s_at | SMCR8 | Up |
| 227379_at | MBOAT1 | Up |
| 227409_at | PPP1R3E | Up |
| 227438_at | ALPK1 | Up |
| 227459_at | --- | Up |
| 227465_at | KIAA0892 | Up |
| 227467_at | RDH10 | Up |
| 227474_at | LOC654433 | Up |
| 227622_at | PCF11 | Up |
| 227639_at | PIGK | Up |
| 227759_at | PCSK9 | Up |
| 227801_at | TRIM59 | Up |
| 227908_at | TBC1D24 | Up |
| 227980_at | ZNF322A | Up |
| 227981_at | CYB561D1 | Up |
| 228008_at | --- | Up |
| 228057_at | DDIT4L | Up |
| 228128_x_at | PAPPA | Up |
| 228186_s_at | RSPO3 | Up |
| 228287_at | ING5 | Up |
| 228320_x_at | CCDC64 | Up |
| 228401_at | ATAD2 | Up |
| 228438_at | LOC100132891 | Up |
| 228531_at | SAMD9 | Up |
| 228624_at | TMEM144 | Up |
| 228707_at | CLDN23 | Up |
| 228708_at | RAB27B | Up |
| 228724_at | --- | Up |
| 228773_at | --- | Up |
| 228899_at | LOC100132884 | Up |
| 228907_at | --- | Up |
| 228923_at | S100A6 | Up |
| 228988_at | ZNF711 | Up |
| 228989_at | C18orf56 | Up |
| 229013_at | LOC145783 | Up |
| 229053_at | SYT17 | Up |
| 229375_at | --- | Up |
| 229762_at | C7orf38 | Up |
| 229779_at | COL4A4 | Up |
| 229810_at | --- | Up |
| 229833_at | --- | Up |
| 229879_at | --- | Up |
| 230172_at | IFI27L1 | Up |
| 230304_at | --- | Up |
| 230314_at | --- | Up |
| 230321_at | --- | Up |
| 230348_at | LATS2 | Up |
| 230588_s_at | LOC285074 /// LOC730268 | Up |
| 230604_at | --- | Up |
| 230642_at | --- | Up |
| 230683_at | ANKRD60 | Up |
| 230761_at | --- | Up |
| 230795_at | --- | Up |
| 230807_at | CCDC151 | Up |
| 230937_at | LOC285835 | Up |
| 230989_s_at | TSSK6 | Up |
| 231078_at | --- | Up |
| 231504_at | CCDC148 | Up |
| 231589_at | LOC100130940 | Up |
| 231725_at | PCDHB2 | Up |
| 231736_x_at | MGST1 | Up |
| 231876_at | TRIM56 | Up |
| 231952_at | --- | Up |
| 231973_s_at | ANAPC1 | Up |
| 232023_at | TMEM67 | Up |
| 232052_at | LOC440944 | Up |
| 232091_s_at | ZDHHC24 | Up |
| 232602_at | WFDC3 | Up |
| 232689_at | LOC284561 | Up |
| 232979_at | --- | Up |
| 233082_at | ZNF630 | Up |
| 233477_at | KLK15 | Up |
| 233616_at | --- | Up |
| 233656_s_at | VPS54 | Up |
| 234302_s_at | ALKBH5 | Up |
| 234488_s_at | GMCL1 /// GMCL1L | Up |
| 234567_at | --- | Up |
| 234707_x_at | IGLV1-44 | Up |
| 234758_at | --- | Up |
| 234838_at | --- | Up |
| 234860_at | --- | Up |
| 234993_at | ABHD13 | Up |
| 235019_at | CPM | Up |
| 235029_at | GINS4 | Up |
| 235076_at | CALCOCO2 | Up |
| 235258_at | DCP2 | Up |
| 235348_at | ABHD13 | Up |
| 235530_at | --- | Up |
| 235533_at | COX19 | Up |
| 235590_at | FAM178A | Up |
| 235607_at | --- | Up |
| 235653_s_at | THAP6 | Up |
| 235658_at | --- | Up |
| 235685_at | --- | Up |
| 235748_s_at | LOC100287052 | Up |
| 235899_at | CA13 | Up |
| 235920_at | --- | Up |
| 235950_at | ZNF688 | Up |
| 235964_x_at | --- | Up |
| 235970_at | LCORL | Up |
| 235974_at | EXOC4 | Up |
| 236155_at | ZCCHC6 | Up |
| 236174_at | --- | Up |
| 236181_at | LOC100132181 | Up |
| 236273_at | NBPF1 | Up |
| 236449_at | --- | Up |
| 236569_at | --- | Up |
| 236690_at | RHBDD1 | Up |
| 236798_at | --- | Up |
| 236922_at | --- | Up |
| 236971_at | --- | Up |
| 237253_at | --- | Up |
| 237449_at | SP8 | Up |
| 237474_at | --- | Up |
| 237601_at | --- | Up |
| 237671_at | --- | Up |
| 237795_s_at | SP2 | Up |
| 237829_at | --- | Up |
| 237883_at | --- | Up |
| 238038_at | --- | Up |
| 238096_at | LOC284023 | Up |
| 238181_at | --- | Up |
| 238244_at | LOC642924 | Up |
| 238264_at | --- | Up |
| 238417_at | PGM2L1 | Up |
| 238456_at | LOC100289230 | Up |
| 238501_at | --- | Up |
| 238509_at | CUL1 | Up |
| 238679_at | MESDC2 | Up |
| 238757_at | DBF4B | Up |
| 238824_at | --- | Up |
| 239115_at | --- | Up |
| 239117_at | --- | Up |
| 239636_at | MCF2L | Up |
| 239682_at | --- | Up |
| 239696_at | --- | Up |
| 239888_at | --- | Up |
| 240031_at | MSRA | Up |
| 240119_at | TEPP | Up |
| 240281_at | --- | Up |
| 240355_at | --- | Up |
| 240592_at | LCORL | Up |
| 241024_at | C6orf147 | Up |
| 241320_at | --- | Up |
| 241353_s_at | --- | Up |
| 241385_at | LARP7 | Up |
| 241721_at | --- | Up |
| 241759_at | --- | Up |
| 241765_at | CPM | Up |
| 241853_at | --- | Up |
| 242260_at | MATR3 | Up |
| 242272_at | ZNF785 | Up |
| 242304_at | WIBG | Up |
| 242307_at | ZNF789 | Up |
| 242329_at | LOC401317 | Up |
| 242521_at | --- | Up |
| 242568_s_at | --- | Up |
| 242584_at | FAM161A | Up |
| 242871_at | PAQR5 | Up |
| 243000_at | CDK6 | Up |
| 243066_at | NPL | Up |
| 243271_at | --- | Up |
| 243356_at | FAM7A3 | Up |
| 243386_at | CASZ1 | Up |
| 243863_at | ZCWPW2 | Up |
| 243868_at | --- | Up |
| 243871_at | LOC100130476 | Up |
| 243931_at | --- | Up |
| 244082_at | --- | Up |
| 244553_at | --- | Up |
| 244687_at | DBT | Up |
| 244816_at | --- | Up |
| 266_s_at | CD24 | Up |
| 33323_r_at | SFN | Up |
| 38241_at | BTN3A3 | Up |
| 38290_at | RGS14 | Up |
| 39248_at | AQP3 | Up |
| 51146_at | PIGV | Up |
| 57082_at | LDLRAP1 | Up |
| 64418_at | SYNRG | Up |
| 65630_at | TMEM80 | Up |
